# Capillary refill time paradoxically decreases in a blood loss shock model

**DOI:** 10.1186/s40635-025-00714-2

**Published:** 2025-01-22

**Authors:** Hugo Gustavsson, Frida Meyer, Sara Fahlander, Birgitta Ölwegård, Hanna Jonasson, Rani Toll, Joakim Henricson, Daniel Wilhelms

**Affiliations:** 1 Department of Emergency Medicine in Linköping, and Department of Biomedical and Clinical Sciences, Linköping University, 582 25 Linköping, Sweden; 2 Department of Biomedical Engineering, Linköping University, Linköping, Sweden

**Keywords:** Capillary refill time, Capillary refill test, Blood loss shock, Lower body negative pressure, Microcirculation

## Abstract

**Background:**

This study aimed to investigate whether changes in capillary refill (CR) time precede macrovascular signs of deterioration in a human model of blood loss shock. The study was conducted at the Department of Emergency Medicine in Linköping, Sweden, and involved 42 healthy volunteers aged 18–45. Participants were randomized into two provocations of applied lower body negative pressure (LBNP): a stepwise escalation protocol and a direct application protocol, to simulate gradual and acute blood loss. The main outcome measure was CR time. Systolic, diastolic, and mean arterial pressures, heart rate, cardiac output, and systemic vascular resistance were measured continuously. CR time was assessed on the finger pulp using a standardized pressure and measured with a polarized reflectance imaging system.

**Results:**

The provocation elicited pre-syncope reactions and clear decrease in blood pressure for all participants, yet two-thirds of the participants in both protocols reacted with shorter CR times at maximum provocation, and the overall median CR time decreased by 0.2 s (Wilcoxon W = − 395.0, range: − 6.3 to 3.2, IQR − 1.3 to 0.1, *P* = 0.0070). Participants with shorter CR times exhibited comparatively greater increases in systemic vascular resistance and a more pronounced decrease in cardiac output.

**Conclusions:**

Our findings reveal that finger CR time paradoxically decreases in a majority of healthy volunteers in a lower body negative pressure model of blood loss, challenging traditional assumptions about the CR test’s reliability as a shock indicator in its present interpretation.

## Introduction

### Background

Hemorrhage is the leading cause of death within the first hour of injury in trauma patients [1–4]. Therefore, it is crucial to have reliable methods to detect central hypovolemia early in bleeding patients [1, 5]. In 1947, Beecher et al. described the capillary refill (CR) test and advocated assessment of CR to be used in conjunction with measurement of blood pressure, pulse quality, thirst, mental state, skin temperature, and skin color as a quick and pragmatic array of tests to assess circulatory status in wounded soldiers [6].

In practice, the CR test is the act of pressing on the skin (most commonly on the finger pulp, but in some guidelines also sternum or forehead) until the blood is displaced from the tissue, resulting in blanching. The CR time is the duration it takes for the tissue to regain its pre-pressure color. Beecher et al. qualitatively graded CR time as “Normal”, “Definite slowing”, “Definite slowing” (again), and “Very sluggish”. These grades were used alongside with results from the other measurements to assess degrees of shock, ranging from “None” to “Severe” [6].

Since 1947, the CR test has become a ubiquitous method for “circulatory assessment” included in many global concepts for triage and the assessment of critically ill adults as well as pediatric patients. [7–9]. The impetus for the widespread adoption of the CR test is somewhat unclear but may originate from 1980 when Champion et al. included the CR test in the “Trauma Score” endorsed by the American College of Surgeons. This was also the first time a quantitative cut-off value for “normal” capillary refill time on the finger pulp was suggested, arbitrarily set at less than two seconds [10, 11]. Normal capillary refill time has since been revised to be below 3 s, and it has also been shown to vary widely with age, temperature and sex [12, 13].

The CR test was not included in the Revised Trauma Score [14], but it has remained popular and extensively used, especially in acute and intensive care settings [7, 8, 15]. Theoretically, the test makes sense—perturbated peripheral perfusion secondary to hypovolemia should lead to a longer refilling time. Additionally, the CR test is very cost-effective, time-efficient, and non-invasive, making it an ideal indicator of circulatory status in theory [13]. However, it remains poorly validated compared to other metrics of circulatory status, and the extent of its widespread use is surprising [13, 16].

In 1991, Schriger et al. performed capillary refill tests on blood donors, before and after donating 450 mL of blood. To our knowledge, this is the only study where the capillary refill test has been tested in a blood loss model. Interestingly, the mean CR time decreased by statistically significant 0.3 s, contrary to the general belief about how capillary refill time should react to blood loss. Schriger et al. hypothesized that this result was due to anxiety regarding the needlestick and did not investigate it further [13].

### Aim

The aim of this study was to investigate if changes in the CR test precede macrovascular signs of deterioration in a human model of blood loss shock.

## Materials and methods

### Participants

This study adhered to the principles outlined in the Declaration of Helsinki regarding ethical standards for research involving human subjects. The study protocol was approved by the Swedish Ethical Review Authority (approval numbers 2020-00140, 2022-04442-02, and 2023-02922-02), and written informed consent was obtained from all participants. Participants had to be between 18 and 45 years old, generally healthy, free from previously known cardiovascular disease, and willing to participate voluntarily. Participants were excluded for blood pressures over 140/90, damaged or tattooed skin on fingertips or forearms, and pregnancy.

**Fig. 1:**
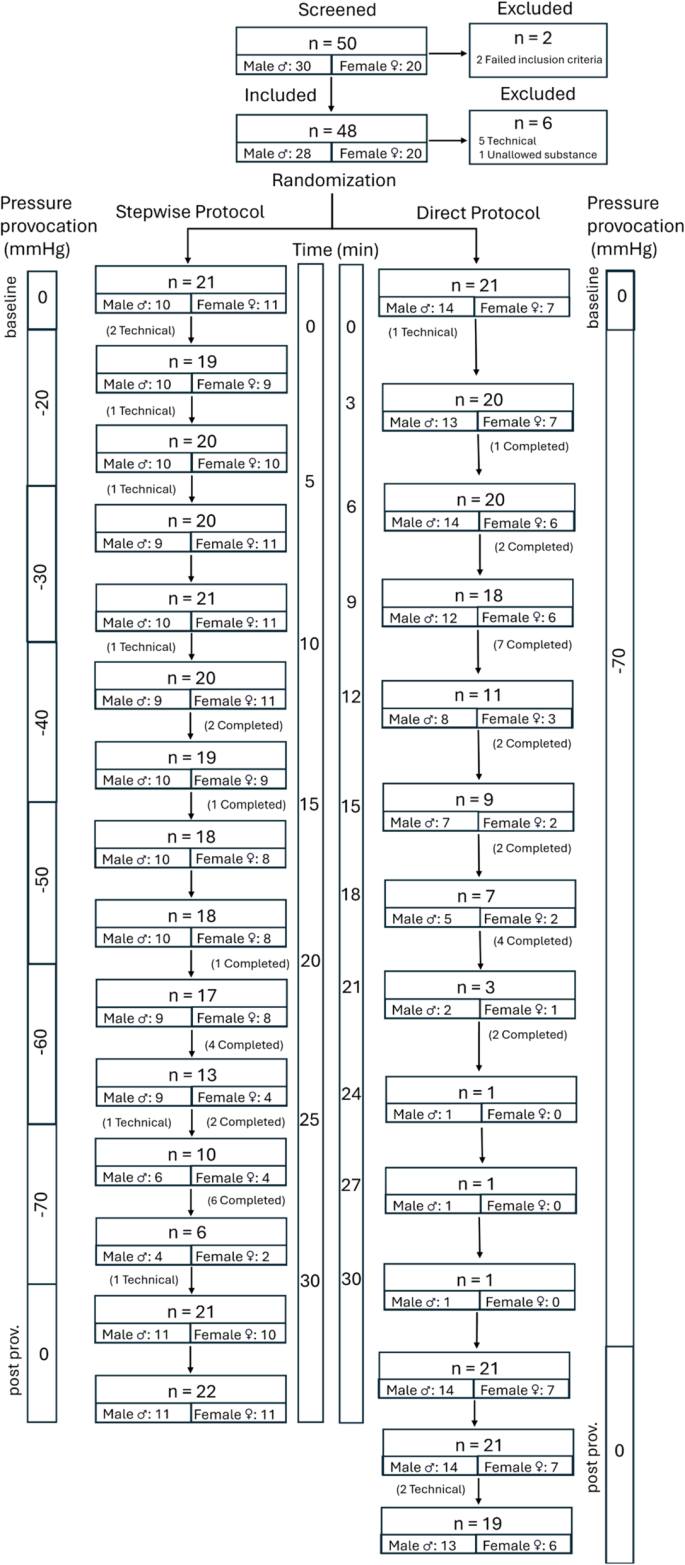
Schematic representation of inclusion and setup of the study. The Stepwise and Direct protocol are represented with flowcharts of data collection occasions, with the number of participants remaining and the male and female distribution on each occasion. The reason(s) for the participant drop-off is stated by; “Technical” in case technical difficulties of data acquiring/analyses resulted in participant drop-off, or “Completed” in case the drop-off is explained by participants exiting, and thereby completing the provocation protocol. Time scale is represented in minutes (min) and level of pressure provocation is represented in millimeters of mercury (mmHg)

Following the informed consent procedure, 50 volunteers were screened (Fig. 1). Participants were randomly assigned to either of two provocation protocols: a stepwise escalation from 0 to − 70 mmHg (− 9.3 kPa) LBNP (“Stepwise”) and a direct application of − 70 mmHg (− 9.3 kPa) LBNP (“Direct”).

The participants were free to end the provocation at any time. Other criteria for terminating the study included participants experiencing pre-syncope symptoms or displaying alterations in vital signs indicating imminent syncope, as determined by an experienced emergency medicine practitioner familiar with the LBNP model. The decision-making process involves weighing together classical subject-reported indicators of pre-syncope such as sudden dryness of the mouth, diaphoresis, nausea and sudden-onset headache. Further, this assessment was assisted by continuously monitoring the trend of vital signs, where pre-syncope is typically characterized by a sudden drop in blood pressure (increasingly negative slope of the curve, rather than an absolute value), and tachycardia. Additionally, the study would conclude upon reaching the protocol’s maximum duration of 60 min at − 70 mmHg (− 9.3 kPa).

### Setup

Prior to the initiation of the experiment, all participants rested for at least 5 min in a supine position in the inactivated LBNP chamber at atmospheric pressure. Baseline measurements were gathered for all included parameters [CR time, systolic and diastolic blood pressure, mean arterial pressure (MAP), heart rate, cardiac output, and systemic vascular resistance (SVR)] before the LBNP provocation started.

### Lower body negative pressure provocation

Lower body negative pressure (LBNP) is a non-invasive and rigorously validated method used to mimic hemorrhage [1, 4]. By decreasing the air pressure around the lower extremities of a human volunteer, LBNP induces central hypovolemia, causing blood to pool in the lower limbs and diverting blood volume away from the thoracic region, thereby replicating the effects of blood loss [1, 4]. This results in reduced venous return to the heart, leading to decreased cardiac output and arterial pressure. Consequently, the body initiates compensatory mechanisms, including increased heart rate, to sustain blood flow to vital organs [17, 18]. At the microcirculatory level, the response has previously been shown to involve a pattern of selective vasoconstriction and vasodilation bringing about a redistribution of the diminished cardiac output in rats [19]. In the cutaneous microcirculation, peripheral resistance rises, and the density of perfused capillaries diminishes, thereby reducing oxygen delivery [17, 20]. This model serves as a valuable tool for investigating physiological responses and adaptations to hypovolemia [1, 4, 17, 18].

During the experiment, participants were subject to pressures between 0 and − 70 mmHg (− 9.3 kPa) below atmospheric pressure, on their lower body (Fig. 1).

Specifically, the LBNP provocation for the Stepwise protocol started at − 20 mmHg (− 2.7 kPa, under atmospheric pressure). The negative pressure was maintained for 5 min before de-escalating in decrements of − 10 mmHg (− 1.3 kPa) until finally reaching − 70 mmHg (− 9.3 kPa, under atmospheric pressure) or if any of the stopping criteria were met. Two CR time measurements were made: one in the beginning and one within the last minute of each pressure, before increasing the negative pressure level. This was the procedure for each pressure level until the second measurement at − 70 mmHg (− 9.3 kPa), where subsequent measurements were made at intervals of 3 min instead, until maximum 60 min.

The Direct protocol started at an immediate pressure of − 70 mmHg (− 9.3 kPa) relative to atmospheric pressure. This pressure was maintained until any stopping criteria were met, or for a maximum of 60 min. CR time measurements were taken just after the negative pressure stabilized, and subsequent measurements were taken at 3-min intervals.

Post-provocation, the participant remained in a supine position in the experimental setup for at least 10 min to ensure recession of any pre-syncope symptoms and normalization of blood pressures and heart rate. During this period, post-provocation CR time measurements were taken. Post-provocation CR time measurements were done at 5 and 10 min in the Stepwise protocol and at 3, 6 and 9 min in the Direct protocol.

### CR test

CR tests were made at the right-hand index fingertip (pulp) and were standardized using a 10 N dynamometer (TickIt 10 N, Hands-On-Science, Järfälla, Sweden). The dynamometer was fitted with a 1.77-cm^2^ round plastic cap to ensure an evenly distributed pressure (0.58 kg/cm^*2*^) over a standardized area. The blanching pressure was applied for 5 s. CR dynamics were assessed using a polarized reflectance imaging system.

### Polarized reflectance imaging

The principles for the polarized reflectance imaging system have been described in detail elsewhere [21]. In short, it is a digital camera-based system that utilizes polarized white light along with a cross-polarized detector to minimize surface glare. The system uses the varying absorption properties of red blood cells and surrounding tissue to derive a value indicative of the concentration of red blood cells in the superficial skin layers. Unlike the surrounding tissue, moving red blood cells exhibit significant light absorption in the green wavelength range (approximately 500–600 nm) and light reflection in the red wavelength range (approximately 600–700 nm). This spectral difference enables visible light spectroscopy through an algorithm that separates the red, green, and blue color matrices and subtracts the green component from the red in each pixel, yielding an output matrix reflecting local red blood cell concentration. Set to video mode, it provides objective means of assessing CR dynamics.

In the present study, the system was set to record at 50 frames per second, at a resolution of 1920 × 1080 pixels.

### Monitoring of vital signs

Systolic and diastolic blood pressure, MAP, SVR and cardiac output were monitored with 1-s resolution using a Finapres (Finapres Finometer Midi, Finapres Medical Systems B.V, The Netherlands). The Finapres sensor was situated on the left-hand middle finger and monitored systolic blood pressure (mmHg), diastolic blood pressure (mmHg), MAP (mmHg), heart rate (beats/min) SVR (Dynes/s/cm^−5^) and cardiac output (L/min).

Brachial blood pressure (systolic, diastolic and MAP) and heart rate (beats/min) were also monitored with a Philips IntelliVue MP-30 (IntelliVue MP-30, Philips Electronics North America Corp., United States) as a back-up system to the Finapres. Medical staff were always present on site to evaluate the subject’s health and vital signs.

### Statistical analysis

Statistical tests were performed using GraphPad Prism version 10.0.2 for Windows (GraphPad Software, Boston, Massachusetts USA, www.graphpad.com).

The parameters heart rate, cardiac output, SVR and CR time were all compared “Baseline” to “Maximum Provocation” (or “Max Prov.”). With an exception for CR time, Baseline was defined as the average of a 5-min period before the provocation, and the Maximum Provocation was defined as the average of the last measurement period before the provocation ended. For CR time, Baseline was defined as the CR test made before the provocation, and the Maximum Provocation was defined as the last CR test made before the provocation ended.

The blood pressure parameters: systolic, diastolic and MAP, were all compared “Baseline” to “Lowest”. For the blood pressure parameters, Baseline was defined as the average of a 5-min period before the provocation, and Lowest was defined as 30 s before the provocation ended.

The distribution of all data was tested with the Shapiro–Wilk normality test. The Wilcoxon matched-pairs signed rank test was used to analyze if there were significant changes in the investigated parameters, and the Mann–Whitney test was used testing significant differences between groups.

## Results

### Participants

The Stepwise protocol group consisted of 21 adults (11 women, 10 men) ranging from 18 to 41 years (mean age: 23 years, SD: 5 years), while the Direct protocol group consisted of 21 adults (7 women, 14 men) between 19 to 32 years (mean age: 23 years, SD: 4 years).

### Blood pressure reaction

Comparing “Baseline” to the “Lowest” blood pressures measured, most notably, systolic blood pressure decreased on average by about 50 mmHg (40%) during the experiment, while diastolic blood pressure decreased on average by around 20 mmHg (25–30%), and MAP decreased by about 30 mmHg (35%) (Table 1).

**Table 1:** Median blood pressure (BP) values with range (min–max), as well as median change in blood pressure and percentual change from the Wilcoxon matched-pairs signed rank test between mean baseline and lowest measured blood pressure during provocation within the protocol groups Stepwise and Direct

			Median (min–max) (mmHg)	Δ (mmHg)	% Δ	p-value	
Stepwise (*n* = 21)	Systolic	Baseline	119 (100–134)	− 47	− 39%	< 0.0001	****
		Lowest	68 (55–118)				
	Diastolic	Baseline	70 (53–89)	− 21	− 30%	< 0.0001	****
		Lowest	47 (26–89)				
	MAP	Baseline	91 (71–105)	− 33	− 36%	< 0.0001	****
		Lowest	55 (35–92)				
Direct (*n* = 21)	Systolic	Baseline	118 (93–141)	− 46	− 39%	< 0.0001	****
		Lowest	71 (55–112)				
	Diastolic	Baseline	71 (52–92)	− 17	− 24%	< 0.0001	****
		Lowest	49 (39–81)				
	MAP	Baseline	86 (67–109)	− 28	− 33%	< 0.0001	****
		Lowest	58 (44–88)				

Both Stepwise and Direct application of LBNP elicited a noticeable blood pressure reaction in most participants. Overall, there were significantly decreased blood pressures (Table 1) of similar magnitude in both protocol groups. During the experiments a general trend was observed, where systolic blood pressure steadily decreased to around 80 mmHg and drastically dropped when subjects reached pre-syncope/syncope state. Diastolic and thus MAP blood pressures also generally decreased in the same manner, however, less dramatically. There was no significant difference in median change in blood pressure changes between the two protocols.

### Capillary refill time

**Fig. 2:**
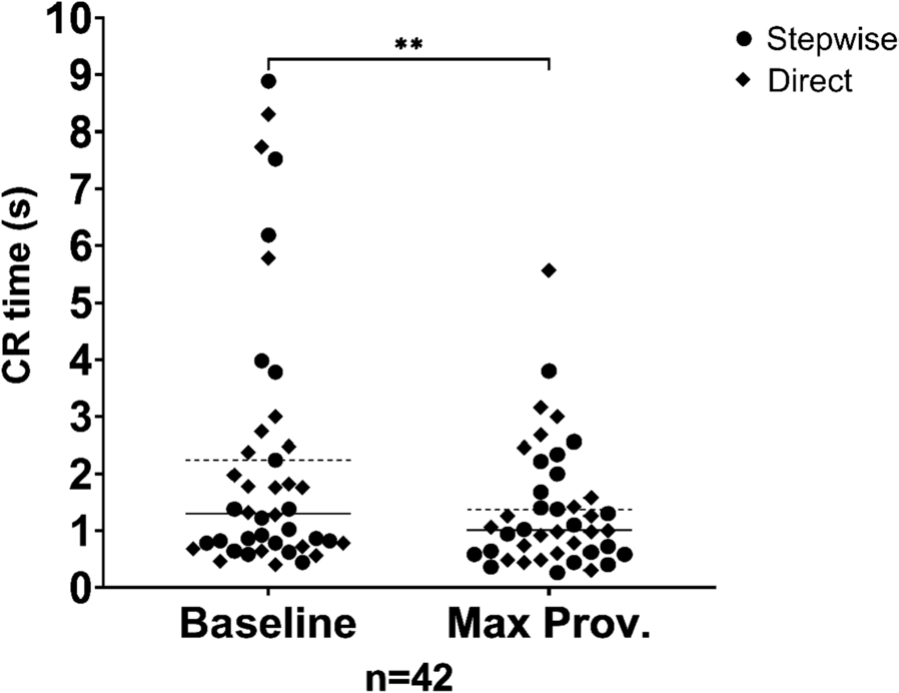
A scatterplot of a Wilcoxon matched-pairs signed rank test (** = *P* ≤ 0.01) between baseline capillary refill (CR) times and CR times at max provocation with data from all participants included (*n* = 42). Median CR times are portrayed with a full line and mean CR times are portrayed with a staggered line. CR times from Stepwise protocol participants are marked with dots and CR times from Direct protocol participants are marked with squares

There was no significant difference in CR time reaction between the two protocols when the change from baseline CR time to max provocation CR time was compared, allowing the protocols to be combined for this analysis. So, between all participants, median CR times became significantly shorter by 0.2 s (line, Fig. 2) when comparing baseline to max provocation (W = − 395.0, range: − 6.3 to 3.2, IQR − 1.3 to 0.1, *P* = 0.0070).

However, all participants did not exhibit shorter CR times. Participants exhibited two distinct, phenotype-like, CR time responses: shorter CR times and longer CR times. About two-thirds of participants reacted with shorter CR times, while one-third reacted with longer CR times. This distribution was consistent across both protocols.

Eight participants (approximately 19% of the trial group) had baseline CR times over 3 s. At max provocation, CR times typically fell below 3 s, often under 2 s. Using a cut-off value of 3 s for CR time, resulted in 3 true positives, 8 false positives, 34 true negatives, and 39 false negatives. Based on these results, the sensitivity and specificity for the CR test with 3 s as cut-off was calculated to 7% and 81%, respectively (Fig. 2).

The CR times are presented in more detail in Appendix 1 (Figs. 4 and 5), where CR times at each time point of the data collection are shown for both protocols. With the exception of the very first time points, a general trend toward shorter CR times can be observed across both protocols. However, it is important to note that the volunteers reached their maximum tolerance to LBNP at different data collection time points.

### Capillary refill time phenotype reactions

**Fig. 3:**
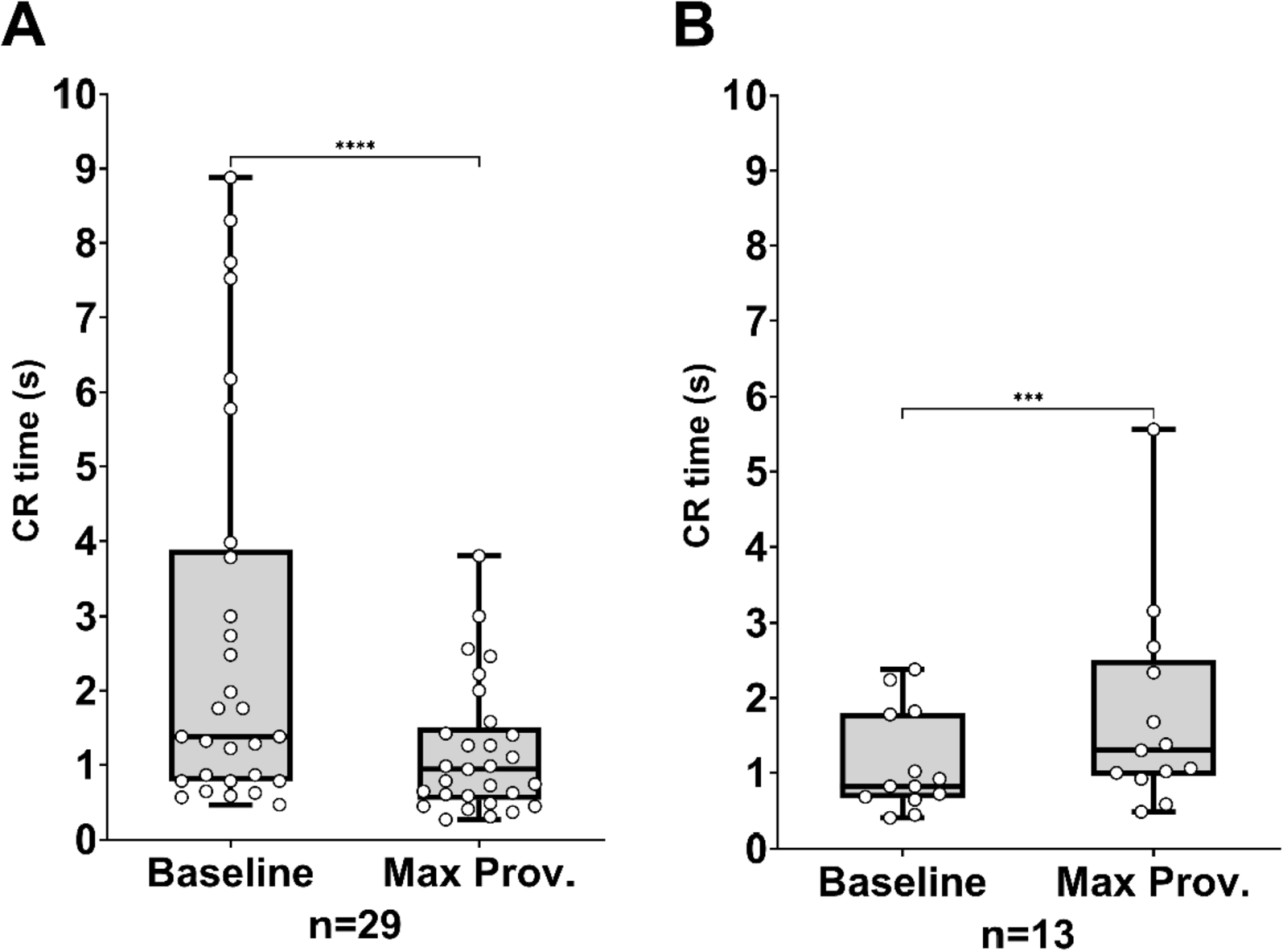
Box plots with distribution (scatter plot, white dots) and range (min–max) whiskers of Capillary Refill (CR) times showing the results of Wilcoxon matched-pairs signed rank tests (*** = *P* ≤ 0.001, **** = *P* ≤ 0.0001) between at baseline and max provocation for participants separated based on shorter (**A**, n = 29) or longer (**B**, *n* = 13) CR time reaction to the provocation. The line in the box plot represents median

**Fig. 4:**
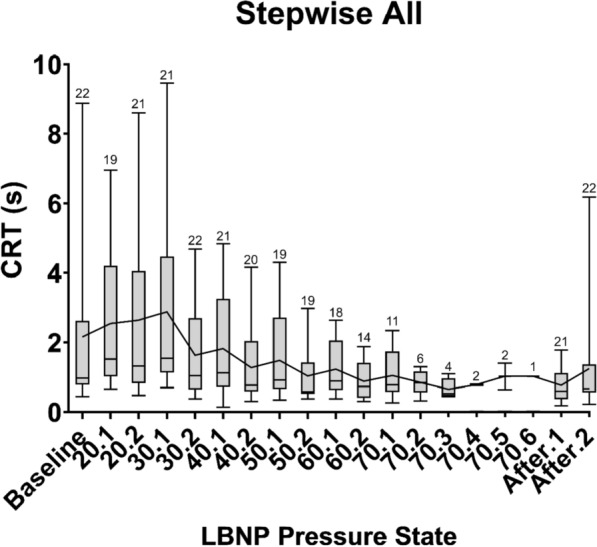
Box plots with range (min–max) whiskers of capillary refill (CR) times (in seconds) for each lower body negative pressure (LBNP) state during the Stepwise protocol (mmHg). Each pressure level (e.g., 20, 30, 40 mmHg, etc.) was maintained for 5 min, during which two CR tests were performed: one immediately after pressure stabilization and another at the end of the 5-min period, just before the pressure was increased. The numbers above the whiskers indicate the number of successful CR measurements (n), with drop-offs representing either unsuccessful tests or protocol completion by the volunteer. The black line within the box indicates the median CR time for each pressure state, while the continuous black line represents the mean CR time across the entire protocol

**Fig. 5:**
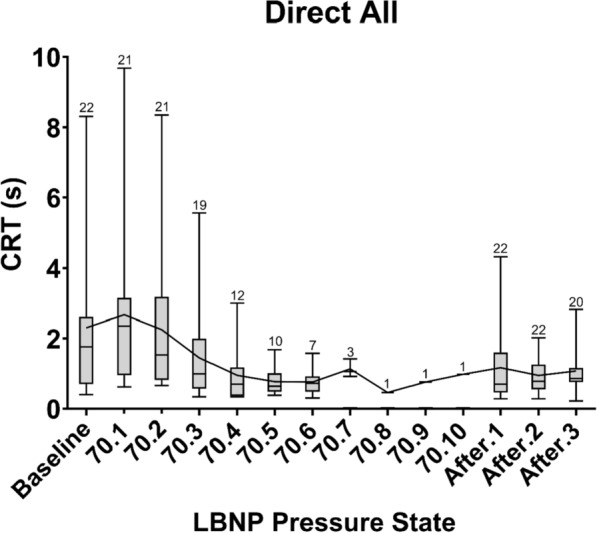
Box plots with range (min–max) whiskers of capillary refill (CR) times (in seconds) for each lower body negative pressure (LBNP) state during the Direct protocol. The pressure was set to 70 mmHg and maintained until protocol completion. A CR test was performed every 3 min (e.g., 70.1, 70.2, 70.3, etc.), starting immediately after pressure stabilization. The numbers above the whiskers represent the number of successful CR measurements (n), with drop-offs reflecting either unsuccessful tests or protocol completion by the volunteer. The black line within the box indicates the median CR time for each pressure state, while the continuous black line represents the mean CR time across the entire protocol

To further investigate the dichotomous CR time response to the provocation, participants were divided into subgroups based on their faster or slower CR time reaction to the LBNP provocation. A Wilcoxon matched-pairs test compared baseline CR times to CR times at max provocation within each subgroup (Fig. 3). In the shorter CR time subgroup median CR time became significantly shorter by 0.6 s (W = − 378.0, range: − 6.3 to 0.0, IQR − 1.–0.2, *P* < 0.0001) (Fig. 3A), while in the longer CR time subgroup median CR time became significantly longer by 0.4 s (W = 91.0, range: 0.1 to 3.2, IQR 0.1–0.9, *P* = 0.0002) (Fig. 3B). Median absolute CR times and the median baseline-corrected percentual time change for both subgroups are detailed in Table 2.

**Table 2:** Median capillary refill (CR) time (seconds), heart rate (beats/minute), systolic pressure (mmHg), diastolic pressure (mmHg), median arterial pressure (MAP) (mmHg), cardiac output (liters/minute), and systemic vascular resistance (SVR) (Dynes/second/cm^−5^), at baseline and max provocation

	CR time		Heart rate		Systolic pressure		Diastolic pressure		MAP		Cardiac output		SVR	
	Sec	% Δ	Beats/min	% Δ	mm. Hg	% Δ	mm. Hg	% Δ	mm. Hg	% Δ	L/min	% Δ	Dynes/ sec. /cm^−5^	% Δ
Shorter CR time (*n* = 29)														
Baseline	1.4		71		119		72		91		5.9		1121	
Max Provocation	0.9	− 42	107	+ 54	71	− 40	52	− 27	60	− 35	4.7	− 34	1293	+ 30
(*p* <)	0.0001		0.0001		0.0001		0.0001		0.0001		0.0001		0.0001	
Longer CR time (*n* = 13)														
Baseline	0.8		67		116		66		86		6.2		1058	
Max Provocation	1.3	+ 47	103	+ 51	64	− 45	44	− 32	51	− 38	4.9	− 21	1223	+ 14
(*p* <)	0.0002		0.0002		0.0002		0.0002		0.0002		0.0012		0.0327	

A Wilcoxon matched-pairs signed rank test was conducted between the baseline and max provocation for each of the parameters and *p*-values according to the test are provided in the table. Median percentual change (%Δ) for each parameter is also provided

The group is separated based on the participants reacting with shorter or longer CR time at max provocation compared to baseline CR times

Further analyses on baseline to max provocation changes for blood pressure, heart rate, cardiac output, and SVR are also summarized in Table 2. Both subgroups showed similar responses in heart rate and blood pressures (systolic, diastolic, and MAP) despite differing CR time reactions. However, there is some difference in SVR and cardiac output. Median SVR increased significantly in both subgroups, with the shorter CR time subgroup exhibiting a larger increase (+ 30%) compared to the longer CR time subgroup (+ 14%). Cardiac output decreased significantly in both subgroups. The shorter CR time subgroup had a larger median decrease (− 1.9 L/min, − 34%) compared to the longer CR time subgroup (− 1.3 L/min, − 21%).

Despite significant within-subgroup changes in SVR and cardiac output, the percentage change in SVR and cardiac output was not statistically significant between the two subgroups.

## Discussion

In this study, we investigated the relation between finger CR time and vital signs in a lower body negative pressure blood loss shock model to test the hypothesis that prolonged finger CR times precede macrovascular changes to impending blood loss shock. We found that, in 42 young healthy volunteers, the CR test as commonly interpreted, could not reliably indicate imminent circulatory collapse. On the contrary, median CR times decreased from 1.3 s at baseline to 1.0 s at maximum blood loss provocation (median decrease: 0.2 s, W = − 395.0, range: − 6.3 to 3.2 s, IQR − 1.3 to 0.1, *P* = 0.0070). Further, two distinct phenotypic responses to the LBNP provocation were found within the study group, where about two-thirds of the group reacted with shorter CR times while one-third of the group reacted with longer CR times. In addition, no significant difference in CR times was found when comparing acute to gradual application of LBNP.

Concerning the advocated for cut-off values for normal CR times [7–10], not only do we see two distinct and different reaction patterns, with about one-third of the participants showing longer and two-thirds showing shorter CR times, but approximately 19% of all participants would be considered near a state of shock at baseline, while around 90% had CR times deemed “normal” even at maximum LBNP provocation. This suggests that a general cut-off value for normal CR time may not be applicable and indicates the need for a cautious approach in both using and interpreting the test itself.

In the two phenotypes, we observed similar changes in cardiac output and SVR. Interestingly, individuals with shorter CR times exhibited a greater decrease in cardiac output than those with longer CR times. Concurrently, those with shorter CR times experienced a larger increase in SVR compared to those with longer CR times. Although the median change between the two subgroups was not statistically significant, the relationship between a larger increase in SVR and a greater loss of cardiac output in the shorter CR time group was consistent across both protocols. However, one must keep in mind that the SVR was calculated from CO and MAP by a mathematical model in the Finapres software (Finapres Finometer Midi, Finapres Medical Systems B.V, The Netherlands), which makes SVR a reductive parameter in this case, meaning that SVR is not fully reliable as an actually measured parameter, but rather a theoretical one.

A possible explanation for the two different responses could be that the CR test is not a measurement solely of capillary perfusion. Rather, the perfusion in the arterioles might make up the larger part of the “visible” perfusion/refill reaction that we measure.

Additionally, we can speculate that a possible mechanism explaining why an increase in peripheral resistance could lead to shorter CR times on the finger pulp could be that, during the CR test, blood is displaced into contracted arterioles under high tension. When the pressure is released, these arterioles rebound more forcefully, causing blood to flow back into the blanched area more quickly. This reasoning could explain a shorter (decreased) CR time while still upholding the hypothesis that peripheral perfusion (such as the perfusion of the skin on the finger pulp) decreases due to contracted vessels in peripheral, non-vital organs during a blood loss. However, this requires further investigation to be fully elucidated, and it does not explain the two CR test phenotypes or how they have similar tolerance to the LBNP provocation despite the differences in CR time.

Ocular (or “naked eye”) assessment of CR time is known to be inherently unreliable, with large inter-individual differences in assessments even between experienced practitioners assessing the same, standardized material [22]. This might be explained by the erythema in the area surrounding the blanching area, skewing the observer’s notion of the baseline color, or that the naked-eye observation might more closely reflect the following hyperemia after blanching, rather than the actual return to baseline. The polarized reflectance imaging method is validated for detecting hemoglobin, which is the main chromophore in the blood detected both optically and by eye. So, whether the refill of the capillaries or the arterioles make up the larger part of the visual refill, a method like polarized reflectance imaging system should be the closest one could come to an objective method of measuring capillary refill.

The capillary refill test, even if it may not be a reliable indicator of hypovolemia, may still offer benefits as experienced medical personnel likely make global evaluations of a patient’s state through observation, and physical examination, where touching the skin may contribute with important information beyond measuring the actual capillary refill time. Further, the traditional application of the CR test seems to perform better in other critical states, like sepsis [23]. These reasons could explain the test’s continued use by many clinicians despite limited supporting evidence for its relevance as an indicator of circulatory status in hypovolemia. Thus, investigating microcirculatory function beyond the scope of the capillary refill test, and beyond hypovolemia, could provide critical insights into assessing a patient’s physiological state, and we should strive to develop more accurate and precise methods that can be used at the bedside. Such methods could harbor great potential to assess the prognosis and response to treatment in a broad range of emergency department and intensive care patients.

### Limitations

Although LBNP is validated as a model for blood loss, compared to bloodletting [1, 4], this blood loss is quite different to what would be encountered in a clinical scenario, with a wound-caused blood loss. Specifically, the LBNP model does not trigger inflammation, coagulation and tissue hypoxia factors in the same manner as injury-related bleeding, which could affect the microcirculatory response in the case of a CR test.

Due to widely varying individual tolerance to the negative pressure provocation, presenting averaged “over time” results for the physiological responses is challenging. With the exception of the very first time points, a general trend toward shorter CR times can be observed across both protocols. This variability complicates the comparison of CR times between subjects, as they are not equally affected despite being exposed to the same absolute levels of LBNP. However, systemic circulation was clearly affected by LBNP, with blood pressure decreasing as provocation and time increased. All subjects eventually experienced a pronounced, sudden drop in blood pressure and reported feelings of pre-syncope, indicating that the model effectively induces significant hemodynamic deterioration. Therefore, comparing responses at baseline and maximum provocation should be sufficient to conclude the effects of LBNP on capillary refill time.

Another limitation of this study is the homogeneity in age and health, as the subjects were relatively young (mean age 23 years, SD 4 years) and in excellent health. To gain more generalizable information, it would be valuable to investigate a broader, more heterogeneous, group of subjects.

## Conclusion

Our findings reveal that finger CR time paradoxically decreases in a majority of healthy volunteers in a lower body negative pressure model of blood loss, challenging traditional assumptions about the CR test’s reliability as a shock indicator in its present interpretation.

## Data Availability

The entire deidentified dataset, data dictionary and analytic code for this investigation are available upon reasonable request, from the date of article publication by contacting Hugo Gustavsson, at email hugo.gustavsson@liu.se.

## Appendix 1

See Figs. 4, 5

## Abbreviations

CR: Capillary refill
LBNP: Lower body negative pressure
MAP: Mean arterial pressure
SVR: Systemic vascular resistance
